# Fungal RNA editing: who, when, and why?

**DOI:** 10.1007/s00253-020-10631-x

**Published:** 2020-05-07

**Authors:** Ines Teichert

**Affiliations:** 1 General and Molecular Botany, Ruhr-University Bochum, 44780 Bochum, Germany; 2 Arbeitskreis für Allgemeine und Molekulare Botanik, Ruhr-Universität Bochum, ND6/166, Universitätsstr. 150, 44780 Bochum, Germany

**Keywords:** RNA editing, Fungi, Development, Adaptation, ADAT, ADAR

## Abstract

**Abstract:**

RNA editing occurs in all kingdoms of life and in various RNA species. The editing of nuclear protein-coding transcripts has long been known in metazoans, but was only recently detected in fungi. In contrast to many metazoan species, fungal editing sites occur mostly in coding regions, and therefore, fungal editing can change protein sequences and lead to modified or new functions of proteins. Indeed, mRNA editing is thought to be generally adaptive on fungi. Although RNA editing has been detected in both, Ascomycota and Basidiomycota, there seem to be considerable differences between these two classes of fungi concerning the types, the timing, and the purpose of editing. This review summarizes the characteristics of RNA editing in fungi and compares them to metazoan species and bacteria. In particular, it will review cellular processes affected by editing and speculate on the purpose of editing for fungal biology with a focus on the filamentous ascomycetes.

**Key Points:**

• *Fungi show various types of mRNA editing in nuclear transcripts.*

• *Fungal editing leads to proteome diversification.*

• *Filamentous ascomycetes may require editing for sexual sporulation*.

• *Wood-degrading basidiomycetes may use editing for adaptation to different substrates.*

## Introduction

The editing of RNA is conserved through all kingdoms of life. Editing occurs in different RNA species, including mRNA, rRNA, and tRNA (reviewed in Knoop 2011). Editing of mRNA can lead to recoding, and thus can induce changes that could also have been coded in the DNA. It can be found in transcripts from mitochondria, plastids, and nuclei.

In general, there are three main types of mRNA editing (reviewed in Knoop 2011). Insertion and deletion of uridines (U) occurs mostly in mitochondrial transcripts, leading to restoration of open reading frames. Cytidin (C) to U editing by deamination occurs in mitochondria and plastids of a wide variety of phyla and in nuclear transcripts of humans, the best characterized being editing of the apolipoprotein B (*apoB*) transcript by APOBEC-1 (Blanc and Davidson 2010; Ichinose and Sugita 2017; Sloan 2017). Plastids show also some reverse editing of U to C. The third type of editing is adenosine (A) to inosine (I) editing, which occurs in metazoan nuclear transcripts (Eisenberg and Levanon 2018). Since I pairs with C, it is interpreted as guanosine (G) in cellular processes like translation (Basilio et al. 1962).

The editing of nuclear protein-coding transcripts was long thought to be absent in fungal species. There are several reasons for this assumption. First, the enzymes catalyzing adenosine deamination in metazoans are not present in fungi. Second, editing was not detected in the yeast *Saccharomyces cerevisiae*, which is often considered a general fungal model, especially outside of the fungal community. Third, at least in ascomycetes, editing occurs only at a specific developmental stage during the lifecycle that has been underinvestigated (Liu et al. 2016, 2017; Teichert et al. 2017).

This review will focus on the editing of nuclear protein-coding transcripts in fungi as well as on the differences of fungal editing to editing in metazoan and bacteria. Besides occurrence and possible mechanisms, it will describe the possible impact of mRNA editing on protein functions and cellular processes and finally discuss the purpose of editing for fungal development.

## Types and occurrence of mRNA editing

Only recently, editing of nuclear protein-coding transcripts has been discovered in fungi. The first descriptions of mRNA editing concerned the filamentous ascomycete *Fusarium graminearum* and the basidiomycete *Ganoderma lucidum* (Zhu et al. 2014; Liu et al. 2016). mRNA editing has not yet been described in the basal fungi. It should be noted that RNA editing sites are commonly detected by calling single nucleotide polymorphisms after mapping RNA-seq reads to reference genomes. Genetically identical strains should therefore be used for genome sequencing and RNA-seq, which is not the case in some published basidiomycete studies, and one has to be cautious about sequencing and mapping errors as well as genetic polymorphisms. Furthermore, most basidiomycetes grow as a Dikaryon for the major part of their life cycle, and transcript variations in dikaryotic strains may thus be derived from allelic variations between the parent genomes.

Both C-to-U and A-to-I editing as well as U-to-C and G-to-A exchanges have been detected in basidiomycetes, but are not enriched in distinct developmental stages. All basidiomycetes investigated for RNA editing so far are from the Polyporales. In these fungi, editing has been reported to diversify transcripts coding for enzymes that are required for the degradation of wood (Zhu et al. 2014; Wu et al. 2018). A comparative study using the wood-decaying fungi *Antrodia sinuosa*, *Daedalea quercina*, *Fomitopsis pinicola*, *Laetiporus sulphureus*, and *Wolfiporia cocos* found an effect of different substrates on editing positions and frequency (Wu et al. 2019).

In contrast, a significant increase of A-to-I mRNA editing is correlated with the formation of sexual fruiting bodies in four genera of filamentous ascomycetes from two different classes, the Sordariomycetes and the Pezizomycetes (Liu et al. 2016, 2017; Teichert et al. 2017). Fruiting bodies of filamentous ascomycetes are distinguished into four different types: apothecia, cleistothecia, perithecia, and pseudothecia (Pöggeler et al. 2006), and RNA editing has hitherto been correlated to the development of both apothecia and perithecia (Liu et al. 2016, 2017; Teichert et al. 2017), although it has been reported to be absent from the apothecia-generating *Botrytis cinerea* (Rodenburg et al. 2018; Bian et al. 2019). It will nevertheless be interesting to investigate this correlation for species generating cleistothecia and pseudothecia. Heterothallic species, needing a mating partner, and homothallic species, being self-fertile, both show an increase of A-to-I RNA editing events when progressing through the sexual cycle.

Since molecular genetic analysis of editing events in the ascomycetes is more advanced than in the basidiomycetes, the following chapters mostly focus on A-to-I editing in ascomycetes, but will give reference to relevant data from basidiomycetes. Table 1 summarizes the characteristics of recoding A-to-I mRNA editing across fungi, metazoa, and bacteria.

**Table 1:** Comparison of recoding A-to-I mRNA editing in nuclear transcripts of humans, cephalopods, fungi, and bacteria

	Humans^1^	Cephalopods^1^	Filamentous ascomycetes^2^	Basidiomycetes^3^	Bacteria^4^
Enzyme	ADAR	ADAR	?	?	ADAT
No. of editing sites (range)	10^2^	10^4^	10^3^–10^4^	10^3^	10^1^
Structure specificity	dsRNA	dsRNA	Loops	?	Loops
Occurence in time/space	Nervous system	Nervous system	During sexual fruiting body formation	No correlation with any developmental stage	Culture density-dependent
Function	Few recoding sites with functional consequences (neurological functions)	Adaptation to different temperatures (neurological functions)	Sexual reproduction?	Substrate specificity of wood-decaying fungi	Tuning of toxin activity

^1^Reviewed in (Yablonovitch et al. 2017); ^2^reviewed here and in (Teichert 2018; Bian et al. 2019); ^3^(Zhu et al. 2014; Wu et al. 2018, 2019); ^4^reviewed in (Bar-Yaacov et al. 2018)

It should be noted that basidiomycetes also show C-to-U mRNA editing, with the same characteristics as A-to-I mRNA editing. *ADAR*, adenosine deaminase acting on RNA; *ADAT*, adenosine deaminase acting on tRNA; *dsRNA*, double-stranded RNA

## Detection of mRNA editing in filamentous ascomycetes

The first A-to-I editing sites discovered in ascomycetes were located in a transcript from *F. graminearum* (Liu et al. 2016). The authors intended to amplify the cDNA of *puk1*, a pseudokinase gene with three introns. The third intron was annotated, because otherwise the ORF would have contained in-frame stop codons. However, cDNA sequencing revealed that the predicted third intron was not spliced, but that two adjacent stop codons had been changed to tryptophan codons in transcripts from perithecia, allowing the translation of a fully functional protein with a complete kinase domain (Liu et al. 2016). This type of editing was termed premature stop codon correction (PSC editing). An overview of how editing of differently located As affect protein functions in fungi is given in Table 2.

**Table 2:** Proposed consequences of A-to-I editing on fungal protein functions

Position of edited A	Vegetative growth and conidiation (no editing)	Fruiting body formation (editing)	
	Functional protein?	Functional protein?	Changes in protein sequence
**Amino acid codon**			
Synonymous codon change	+	+	–
Non-synonymous codon change	+	+ Modified function?	Protein with one to few amino acid exchanges Single amino acid variation (SAAV)
**Stop codon**			
Premature stop codon in pseudogenes	–	+	Longer protein version with complete domains and motifs Premature stop codon correction (PSC)
Translational stop codon in complete genes	+	+ Additional/modified function?	Longer protein version with additional domains and motifs Stop loss (SL)

Tens of thousands of editing sites were then identified in *F. graminearum* and *F. verticilloides*, *Neurospora crassa* and *N. tetrasperma*, *Sordaria macrospora*, and the Pezizomycete *Pyronema confluens* by RNA-seq. A database of A-to-I RNA editing events has been implemented, termed FairBase (a fungal A-to-I RNA editing database), listing editing events from the six ascomycetes (Liu et al. 2019). When mapping RNA-seq data to the reference genome and searching for base changes, A-to-G exchanges are indicative of A-to-I editing, because of the pairing of I to C as mentioned above. These analyses showed that fungal editing sites occur mostly in coding regions, which is in contrast to many metazoan species including humans, where editing is prevalent in non-coding regions, but similar to cephalopods, where tens of thousands of editing sites occur in coding regions (Yablonovitch et al. 2017; see Table 1). Besides PSC editing, As in amino acid codons are targeted in fungi, leading to synonymous or non-synonymous codon changes (Table 2). Interestingly, editing also targets stop codons of correctly annotated functional genes, often changing a *UAG* into a *UGG* tryptophan codon, causing translation to run on into the 3′-UTR (stop loss (SL) editing, Table 2) (reviewed in Teichert 2018).

Besides transcriptomic analysis, proteogenomics was applied to give peptide-level evidence for protein sequence changes caused by editing in *S. macrospora* (Blank-Landeshammer et al. 2019). Importantly, standard proteomics approaches match mass-spectrometry-derived peptide sequences to a peptide library generated from annotated genes. However, these approaches only detect peptides that are to be expected because of the annotation of the genome, and thus are biased. In proteogenomics, peptides are matched to a 6-frame translation of the genome, making the analysis independent of annotation errors and allowing the detection of peptides from C-terminally elongated proteins due to SL editing. By proteogenomics, evidence for 113 single amino acid variations (SAAVs, Table 2) and 15 stop loss events stemming from RNA editing was detected at the protein level (Blank-Landeshammer et al. 2019). Still, proteogenomics faces the same problems as any peptide-based proteomics approach. Some peptides from proteolytic cleavage of protein extracts cannot be detected in a mass spectrometry experiment due to the experimental setup, the peptide, or the general complexity of biological samples (Ly and Wasinger 2011).

Similar to ascomycetes, different consequences of editing have been reported in basidiomycetes. In some of the analyzed species, non-synonymous codon changes were enriched over synonymous codon changes. However, fewer events of SL editing were detected than in the ascomycetes, and PSC editing was not reported on (Zhu et al. 2014; Wu et al. 2018, 2019).

## Editing mechanisms

A-to-I mRNA editing in metazoan species is mediated by adenosine deaminases acting on RNA (ADARs, Table 1). These deaminases contain an adenosine deaminase domain as well as differing numbers of double-stranded RNA (dsRNA)-binding motifs (Nishikura 2016). They bind to ds RNA and edit bases in one strand using the second strand as template. For example, the transcript for glutamate receptor GRIA2 becomes edited at the so-called Q/R site, and editing requires the formation of a dsRNA between the corresponding exonic sequence and an intronic editing site complementary sequence (ECS) (Higuchi et al. 1993). In metazoan, different ADARs are used for different editing purposes. Human ADAR1 isoforms edit non-coding repeat regions, while ADAR2 is required for recoding editing, which rarely exists in humans (Table 1). Cephalopods likewise contain ADAR1 and ADAR2, but show increased editing in coding regions compared to humans (Table 2). For squid, it has been shown that ADAR2 contains more dsRNA binding domains than its human ortholog and more than ADAR1, which may account for the more frequent editing of coding regions (Yablonovitch et al. 2017). These data show that editing regulation can be rather diverse. As mentioned above, fungi do not have ADAR orthologs and thus must use a mechanism different from the one in metazoa.

A-to-I editing does not only occur in mRNA, but also in tRNA. Seven to eight cytoplasmic tRNAs and one tRNA in plastids and bacteria show inosine at the wobble position of the anticodon loop (Sprinzl et al. 1998; Gerber and Keller 1999). A-to-I editing in tRNAs is mediated by adenosine deminases acting on tRNA (ADATs) (Torres et al. 2014). These enzymes recognize the overall tRNA structure, although bacterial Tad1 is also able to deaminate a mini-substrate containing only the stem-loop structure of the anticodon arm. Since editing sites of ascomycetes are located mostly in hairpin loops, Liu et al. (2016) raised the question if fungal ADATs might be able to edit mRNAs. Indeed, it has been shown that mRNA editing in bacteria is mediated by an ADAT homolog (Table 1) (Bar-Yaacov et al. 2017). If ADATs act similarly in fungi is still under debate. *F. graminearum*, *N. crassa* and *S. macrospora* each contain three *adat* genes homologous to *S. cerevisiae tad1*, *tad2*, and *tad3* (Liu et al. 2016, 2017; Teichert et al. 2017). However, *tad1* deletions show no phenotype, and *tad2* and *tad3* could not be deleted in *F. graminearum*, probably due to the essential nature of tRNA A-to-I editing (Liu et al. 2016).

In contrast to ascomycetes, basidiomycetes do not specifically show A-to-I editing, but also C-to-U, U-to-C, and G-to-A conversions (Zhu et al. 2014; Wu et al. 2018, 2019). Here, further enzymatic activities might be required. Thus, the nature of the fungal editing mechanism(s) is still under debate.

## Evolutionary aspects of mRNA editing

The evolution of editing in ascomycetes has been reviewed extensively in Bian et al. (2019). As mentioned above, fungal editing sites occur mostly in coding regions and lead to different consequences for proteins (Table 2), and these different types of editing may differ from the evolutionary point of view. In general, editing may confer adaptive advantages, especially when conserved or essential genes or sites are concerned that cannot be targeted by mutation at the DNA level (Bian et al. 2019). It was shown for *N. crassa* that non-synonymous editing sites are under positive selection and tended to affect genes with higher functional constraint (Liu et al. 2017). The authors also compared editing sites that were conserved between the *Fusarium* and *Neurospora* species and found that the conserved sites show higher editing levels and had a higher probability to cause SAAVs. SL editing appeared to be under even stronger positive selection (Liu et al. 2017). Interestingly, PSC editing is comparable to mRNA editing in organelles in that it repairs otherwise non-functional transcripts (Bian et al. 2019). Thus, ascomycetes are more comparable to cephalopods than to humans, because like in cephalopods editing seems to be adaptive (Table 1) (Liscovitch-Brauer et al. 2017).

## Cellular processes affected by editing

As outlined above, editing at conserved sites is considered generally adaptive and most likely to alter protein functions. The analysis of the corresponding gene set in the analyzed Sordariomycetes led to the conclusion that specific cellular processes may be affected (e.g., Liu et al. 2017; Wang et al. 2019), among them (epi-) genetic phenomena, autophagy, and cellular signaling.

Several phenonema occur specifically during sexual development in a number of ascomycetes, for instance, repeat-induced point mutation (RIP), and meiotic silencing by unpaired DNA (MSUD) (Gladyshev 2017). RIP introduces point mutations into repetitive sequences and occurs premeiotically. MSUD occurs during meiosis and is used for silencing of unpaired genes. In both *F. graminearum* and *N. crassa*, genes involved in RIP and MSUD seem to be highly affected by RNA editing (Wang et al. 2016). The *rid-1* cytosine methyltransferase gene is responsible for methylation of RIP targets in *N. crassa*, and *F. graminearum* as well as *F. verticilliodes rid-1* homologs require PSC editing to become functional. *F. graminearum* genes required for MSUD show non-synonymous editing events of their transcripts. It has been hypothesized that RIP and MSUD are regulated in a stage-specific fashion by RNA editing (Wang et al. 2016).

Another process affected by RNA editing is autophagy. Autophagy is a degradation process in eukaryotes that removes damaged or surplus cellular machinery, thereby recycling nutrients, and is vital for proper development (Hale et al. 2013). Several autophagy genes (*atg*) have been shown to be required for sexual fruiting body formation in filamentous ascomycetes (Khan et al. 2012; Voigt and Pöggeler 2013). Interestingly, *atg* transcripts are highly edited in both *F. graminearum* and *N. crassa*. In total, 10 editing sites in 9 *atg* genes were conserved between both species (Wang et al. 2019). Therefore, the authors hypothesized that editing may be necessary for adaptation of autophagy to specific requirements during sexual development.

This hypothesis may be valid for other cellular processes. Liu et al. (2017) observed that many *N. crassa* genes whose transcripts undergo SL editing had previously been characterized as developmental genes involved in different stages of fruiting body formation, and the same is true for *F. graminearum* and *S. macrospora* (Liu et al. 2016; Teichert et al. 2017). Besides genes involved in histone modification and chromatin remodeling, these genes encode developmental transcription factors and pheromone-receptors, among others. Strikingly, the dataset also contains genes coding for conserved eukaryotic signaling complexes, such as the striatin-interacting phosphatase and kinase (STRIPAK) complex, and the COP9 signalosome (Liu et al. 2017; Teichert et al. 2017). Both are multi-protein complexes that regulate conserved cellular functions like cytokinesis and protein degradation, but both have also been shown to regulate fruiting body formation in filamentous ascomycetes (reviewed in Braus et al. 2010; Kück et al. 2019). Hypothetically, mRNA editing may change the composition, localization, and function of these conserved protein complexes and thereby specify their function for the specific purpose of generating progeny in generative tissues.

Thus far, the analysis of editing sites has been restricted to those that correct premature stop codons, leading to functional proteins, or change stop codons of functional ORFs into tryptophan codons, leading to C-terminally elongated proteins with supposedly additional functions (Liu et al. 2016; Table 2; Liu et al. 2017). However, a number of editing events in coding regions lead to either non-synonymous or synonymous amino acid codon changes. Non-synonymous changes that lead to SAAVs could modify protein functions due to altered physicochemical properties (Liu et al. 2016; Blank-Landeshammer et al. 2019). Synonymous codon changes are often considered negligible. Still, recent data suggest a huge effect of these silent mutations on co-translational protein folding (Yu et al. 2015). Another consequence of RNA editing that has not yet been investigated is the impact on the mRNA itself. Exchange of As to Is induces new pairing situations and may thus lead to structural changes of the mRNA. This in turn may cause a difference in stability as well as binding to other RNAs or proteins.

## On the purpose of fungal editing in the ascomycetes

Regarding the purpose of mRNA editing in fungi, one has to consider the timing of the editing process and the function and transcriptional expression of genes whose transcripts become edited. In general, transcripts affected by editing tend to be upregulated or even specifically expressed during fruiting body formation. For *S. macrospora*, it was shown that expression of these transcripts tends to be downregulated in mutant pro1 and thus is controlled by a developmental transcription factor (Liu et al. 2016, 2017; Teichert et al. 2017).

Transcriptomic and proteomic data indicate that editing occurs during the formation of sexual fruiting bodies and the generation of ascospores (Liu et al. 2016, 2017; Teichert et al. 2017; Blank-Landeshammer et al. 2019). Specifically, in *F. graminearum* and *N. crassa*, A-to-I editing was detected in RNA-seq data from fruiting bodies, but not from conidia and vegetative mycelium (Liu et al. 2016, 2017). Comparison of RNA-seq data from 3, 4, 5, and 6 days post fertilization (dpf) *N. crassa* perithecia revealed that editing frequency increases over time and that different sites are edited at different time points (Liu et al. 2017). For *P. confluens*, it was also shown that editing frequency increases in sexually developing cultures, while in vegetative cultures, it did not. In *S. macrospora*, a significant increase of A-to-G base changes was detected in microdissected protoperithecia, but not in vegetative hyphae (Teichert et al. 2017). Furthermore, quantitative analysis of SAAV- and SL-specific peptides by mass spectrometry showed that they occurred only in cultures in late stages of development (Blank-Landeshammer et al. 2019).

Data from *N. crassa* and *S. macrospora* indicate that editing occurs before the onset of meiosis, but increases during ascus and ascospore development (Liu et al. 2017; Teichert et al. 2017). In *N. crassa*, low-level editing was detected in 3 dpf perithecia without ascus differentiation. Similar results were obtained from 5 dpf perithecia of mutant Δstc-1 lacking asci completely. Mid-level editing was found in 5 dpf perithecia of mutant Δsad-1 that arrests ascus development in meiotic prophase (Liu et al. 2017). In *S. macrospora*, wild type protoperithecia showed editing, but protoperithecia of developmental mutants pro1 and Δnox1 did not (Teichert et al. 2017). Both mutants have a developmental arrest after protoperithecia formation and do not form asci (Masloff et al. 1999; Dirschnabel et al. 2014). Editing may thus take place in distinct cell types at specific time points during development.

Concerning function, the deletion of genes whose transcripts are affected by editing tend to have defects in the formation, morphology, or discharge of ascospores, but rarely in the formation of the fruiting bodies themselves. For example, deletion of the autophagy gene *atg11* and the kinase gene *puk1* in *F. graminearum* resulted in a defect in ascospore discharge (Liu et al. 2016; Wang et al. 2019). Deletion of the *F. graminearum AMD1* gene, coding for a major facilitator superfamily domain protein, resulted in defects in ascus wall formation, ascospore discharge, and germination of ascospores inside perithecia (Cao et al. 2017). Fewer spores mature and are released in a mutant lacking *FGRRES_01649* encoding a Rho GTPase (Liu et al. 2016). In *N. crassa*, deletion of *NCU10184* as well as *stk-21* resulted in delayed ascospore formation and the generation of abnormal asci. The *stk-21* mutant further showed a weakened ascospore wall and morphologically abnormal asci (Liu et al. 2017). In the ΔNCU11109 mutant, more than half of the perithecia did not contain asci. Deletion mutants of *NCU07992* and *NCU04187* showed no perithecia formation (Liu et al. 2017).

Do the editing sites have biological functions? It has been shown for the *AMD1* and *puk1* genes that non-editable alleles are unable to complement the phenotype of the corresponding deletion mutant, whereas the edited alleles do complement (Liu et al. 2016; Cao et al. 2017). In *N. crassa*, non-editable alleles of *stk-21* and *NCU10184* failed to restore normal ascospore formation in the corresponding deletion strains (Liu et al. 2017). All of these analyzed genes show PSC editing of their transcripts and here, only the edited allele seems to be functional. However, for distinct SL editing events, both the short and long protein versions may have functions in ascospore formation and discharge, since strains expressing only the short or only the long allele both were still compromised in sexual development (Teichert unpublished).

Taken together, these observations lead to the conclusion that editing may be required for ascospore formation. However, this hypothesis cannot be tested until the fungal editing mechanism has been described and can be specifically inhibited. Anyway, deliberately switching on mRNA editing would give fungi the opportunity to diversify their proteome using their given genomic content at a very specific developmental stage.

Why would fungi need this diversification of their proteome? Little is known about the molecular determinants of ascospore formation. Once the ascus is formed and meiosis has been completed, membranes have to be formed to surround the nuclei, and the cytoplasm including organelles, RNA, and proteins has to be sorted into the spores in a regulated manner. It seems reasonable that specifically adapting key cellular functions might aid in this process. However, *Schizosaccharomyces pombe* also generates ascospores within asci, but no mRNA editing was detected in meiotic samples (Teichert et al. 2017).

Another advantage of RNA editing prior to ascospore formation might be that it enables the fungus to provide its progeny with either mRNAs or proteins that might give an advantage in the environment. However, it needs to be proven that different isoforms of proteins or differently edited transcripts occur in the mature ascospores.

## Conclusions

RNA editing in fungi is still a new phenomenon from the researching perspective. In ascomycetes, it may be required for sexual spore formation, while in basidiomycetes, it may enable growth on different substrates. However, the question about the distribution of mRNA editing in the fungal kingdom remains open. Yet, sampling more species might give a hint about the evolution of editing in fungi. Further, the fungal editing enzyme(s) and regulators need to be identified. It has been shown that editing indeed changes protein sequences, but the effect of editing on protein functions is not fully understood and especially in the basidiomycetes, diversified protein functions have yet to be tested. Taken together, more research is needed to understand the mechanism(s) and the role(s) of mRNA editing for the kingdom fungi.
